# Paranemic Cohesion of DNA under Isothermal Conditions

**DOI:** 10.1021/jacsau.6c00130

**Published:** 2026-03-06

**Authors:** Lauren A. Anderson, Akul Patel, Bharath Raj Madhanagopal, Hannah Talbot, Nada Kabbara, Ken Halvorsen, Arun Richard Chandrasekaran

**Affiliations:** † Department of Nanoscale Science and Engineering, University at Albany, State University of New York, Albany, New York 12222, United States; ‡ Department of Biological Sciences, University at Albany, State University of New York, Albany, New York 12222, United States; § The RNA Institute, University at Albany, State University of New York, Albany, New York 12222, United States

**Keywords:** DNA nanotechnology, isothermal assembly, paranemic crossover DNA, PX DNA, DNA nanostructures, counterions

## Abstract

Recent progress in DNA nanotechnology has shown the isothermal assembly of several DNA nanostructures. Isothermal assembly allows DNA nanostructure construction in a variety of ions while simplifying DNA nanotechnology by avoiding the need for thermal cyclers and expands utility by enabling attachment of guest biomolecules on DNA nanostructures at ambient or physiological temperatures. The paranemic crossover (PX) DNA motif has been used in the construction of DNA nanostructures, paranemic cohesion has been used to connect DNA structures as an alternate to sticky end cohesion, and PX DNA has also been implied to have a biological role in homology recognition. In that context, here we demonstrate the successful isothermal assembly of the PX DNA motif in magnesium (Mg^2+^), calcium (Ca^2+^), and strontium (Sr^2+^) at 20 and 37 °C. Using isothermal titration calorimetry, we show that interhelix hybridization of half-PX molecules is favored at higher temperatures, with a heat capacity (ΔC_p_) of −1.9 kcal/mol·K. To demonstrate a key advantage of isothermal assembly, we show that PX molecules can be designed to contain thrombin-specific aptamers for binding one or two thrombin molecules site specifically in an entirely isothermal procedure. Our work extends isothermal assembly and the use of different counterions for complex DNA motifs while demonstrating the attachment of guest molecules at constant temperatures.

## Graphical abstract

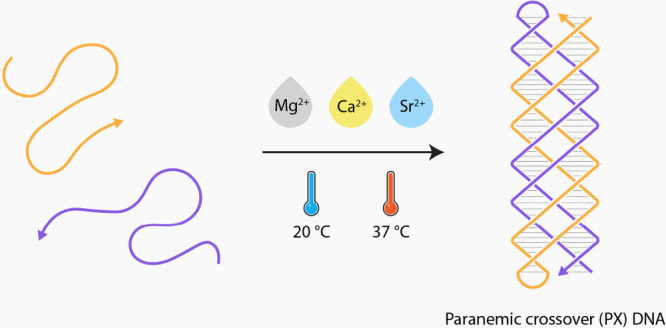

Paranemic crossover (PX) DNA is a multistranded, multicrossover motif used in the assembly of DNA objects,^1,2^ devices,^3,4^ arrays,^5,6^ and origami.^7^ PX DNA consists of two adjacent and connected double helical DNA domains, formed by creating crossovers between strands of the same polarity at every possible point between the two helices (Figure 1a).^8,9^ Each duplex domain contains alternating wide or narrow groove separation flanking the central dyad axis of the structure, with the helical repeat of a PX molecule (∼22 bp) being twice that of conventional B-DNA. PX DNA is typically assembled in magnesium-containing buffers by using a thermal annealing protocol. We, and others, have shown isothermal assembly of several DNA nanostructures by incubation at constant temperatures instead of thermal annealing.^10–17^ Further, we recently demonstrated that DNA nanostructures can be assembled in different counterions instead of the typically used magnesium.^10,18,19^ Assembly in other counterions provides enhanced biostability of DNA nanostructures^19^ and higher assembly yields,^20^ making DNA nanostructure assembly suitable for different application contexts. In addition, a variety of counterions also allows isothermal assembly of DNA nanostructures at constant temperatures, eliminating the need for thermal cyclers in DNA nanostructure assembly.^10,14^ In this work, we expand the isothermal assembly method to PX DNA, showing assembly in magnesium (Mg^2+^), calcium (Ca^2+^), or strontium (Sr^2+^). Exploring the isothermal assembly of PX DNA could add to its biological implications. PX DNA has been suggested to be involved in homology recognition in cells^21^ and in structure-dependent binding of proteins *Escherichia coli* DNA polymerase I and T7 endonuclease to variations of the PX structure.^22,23^ In addition, PX DNA has been amplified through enzymatic replication^24^ and *in vivo* cloning in *E. coli* cells.^25^ While PX DNA assembly in Mg^2+^ has been shown experimentally and *in silico*, there are no experimental reports of PX DNA formation in ions other than Mg^2+^.

**Figure 1:**
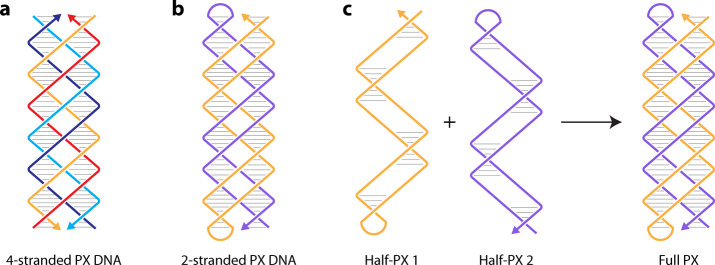
**Paranemic crossover DNA** *.* (a) Schematic of a 4-stranded PX DNA motif with alternating 6 and 5 base pairs in the half turns. (b) A 2-stranded PX DNA containing a loop at two ends. (c) Two half-PX molecules can connect to form a full PX structure via PX cohesion.

The PX structure we chose contains 6 nucleotides in the major groove and 5 nucleotides in the minor groove (PX 6:5), known to be the most stable among PX molecules with helical parameters close to those of the B-DNA double helix.^9,26–29^ While PX DNA is typically constructed using four strands, the structure can also be realized by two “half-PX” molecules that are partially base-paired and contain a loop at one end (Figure 1b).^30^ The unpaired regions of the two half-PXs are designed to be complementary so that the two duplexes wind around each other in a right-handed helix, resulting in a PX structure (Figure 1c). The backbones of the two-component duplex domains are not linked and thus can be separated from each other without the need for a strand break. This character of PX DNA has allowed its use in connecting different DNA motifs, providing paranemic cohesion of DNA molecules.^30^

We first tested the effect of Mg^2+^, Ca^2+^, and Sr^2+^ concentrations on isothermal PX DNA assembly (Figure 2a and sequences in Table S1). We assembled the structure in tris-acetate-EDTA (TAE) buffer containing 12.5 to 125 mM of the counterion followed by incubation at room temperature (20 °C) or physiological temperature (37 °C) for 1 h. Nondenaturing polyacrylamide gel analysis showed assembly of the structure at both temperatures and at all tested ion concentrations (Figure 2b and Figure S1). The assembly yields were estimated by a densitometric analysis of the gel and normalized to the yields obtained when the structure was annealed in 12.5 mM Mg^2+^. We observed an increase in the assembly yield with increasing Mg^2+^ concentration, with relative assembly yields at 37 °C being slightly higher than those at 20 °C. When isothermally assembled, the highest assembly yields in 125 mM Mg^2+^ were ∼ 90% and ∼ 120% for 20 and 37 °C, respectively, when compared to the annealed control. Assembly yield in 12.5 mM Ca^2+^ at 37 °C (∼94%) was comparable to the annealed structure while isothermal assembly in 25, 50, and 125 mM Ca^2+^ (120–132% normalized yields) surpassed the yield of the sample annealed in Mg^2+^. Normalized assembly yield in Sr^2+^ ion was slightly lower compared to those of Mg^2+^ and Ca^2+^, with ∼ 81% at 20 °C and ∼ 93% at 37 °C in 125 mM ion concentration. We confirmed that these ions also yielded proper assembly of the PX molecule by using a thermal annealing protocol (Figure S2). Analysis of PX DNA assembly in 125 mM counterions over different time periods showed an increase in assembly yield over time at 20 °C, with the highest yields at the maximum time point tested (2 h), while 30 min incubation yielded effective assembly at 37 °C (Figure 2c and Figure S3). This increase in assembly yield with time was similar in all the counterions tested.

**Figure 2:**
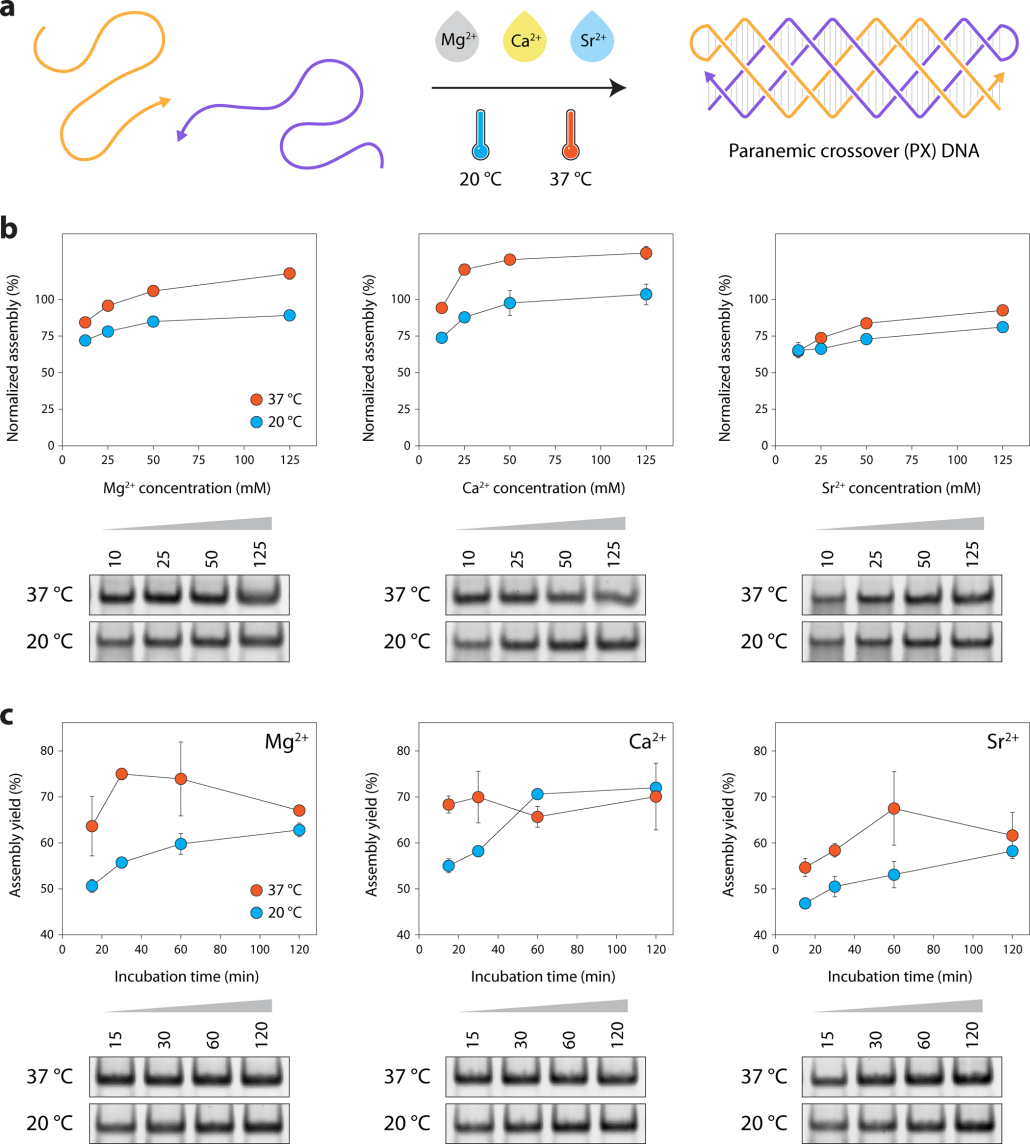
***Isothermal assembly of PX molecules***. (a) A 2-stranded PX molecule assembled from two 80-nt long oligonucleotides containing a 4-thymine loop. (b) Assembly of the PX molecule in TAE buffer containing different concentrations of Mg^2+^, Ca^2+^, or Sr^2+^ at a constant temperature of 20 or 37 °C. Assembly yields are normalized to the yield of the structure assembled in TAE buffer containing 12.5 mM Mg^2+^ using a thermal annealing protocol. (c) Assembly of the PX molecule at different incubation times in buffer containing Mg^2+^, Ca^2+^, or Sr^2+^. Data are presented as the mean ± standard deviation (SD) from three replicate experiments.

While these divalent ions showed successful assembly of the PX molecule under these temperatures, we did not observe proper assembly in monovalent ions Na^+^, Li^+^, and K^+^ even at ion concentrations as high as 250 mM (Figure S4). The complexity of the structure, including the topology of two loop-containing strands and the continuous crossovers in the PX structure, may play a role in effective isothermal assembly, as we have previously showed assembly of a similar-sized double crossover DNA motif in these monovalent ions.^10,19^ We note that the PX 6:5 molecule has been simulated in the presence of Na^+^ before,^29^ and the stretch modulus of the structure was higher in Mg^2+^ compared to Na^+^ conditions. Since the PX structure is more rigid compared to duplexes and double crossover motifs,^29,31^ divalent ions might provide better screening for the negative charges between the helical domains compared to monovalent ions.

Next, we determined the thermodynamic parameters involved in PX cohesion. The thermodynamics of PX DNA has previously been studied by investigating the unfolding of a thermally annealed PX structure using differential scanning calorimetry and UV-spectroscopy.^32^ Our successful isothermal assembly of PX DNA allows us to directly study the formation of the structure, and the 2-stranded PX DNA is a suitable model nanostructure for analysis by isothermal titration calorimetry (ITC) as it provides a mechanism to measure thermodynamic parameters of bimolecular interactions without labeling. We note that within each half-PX, there are regions with intrahelix hybridization of base pairs. Combining the two half-PX molecules results in the formation of additional interhelix base pairs, the energies of which are obtained in this study (Figure 3a).

**Figure 3:**
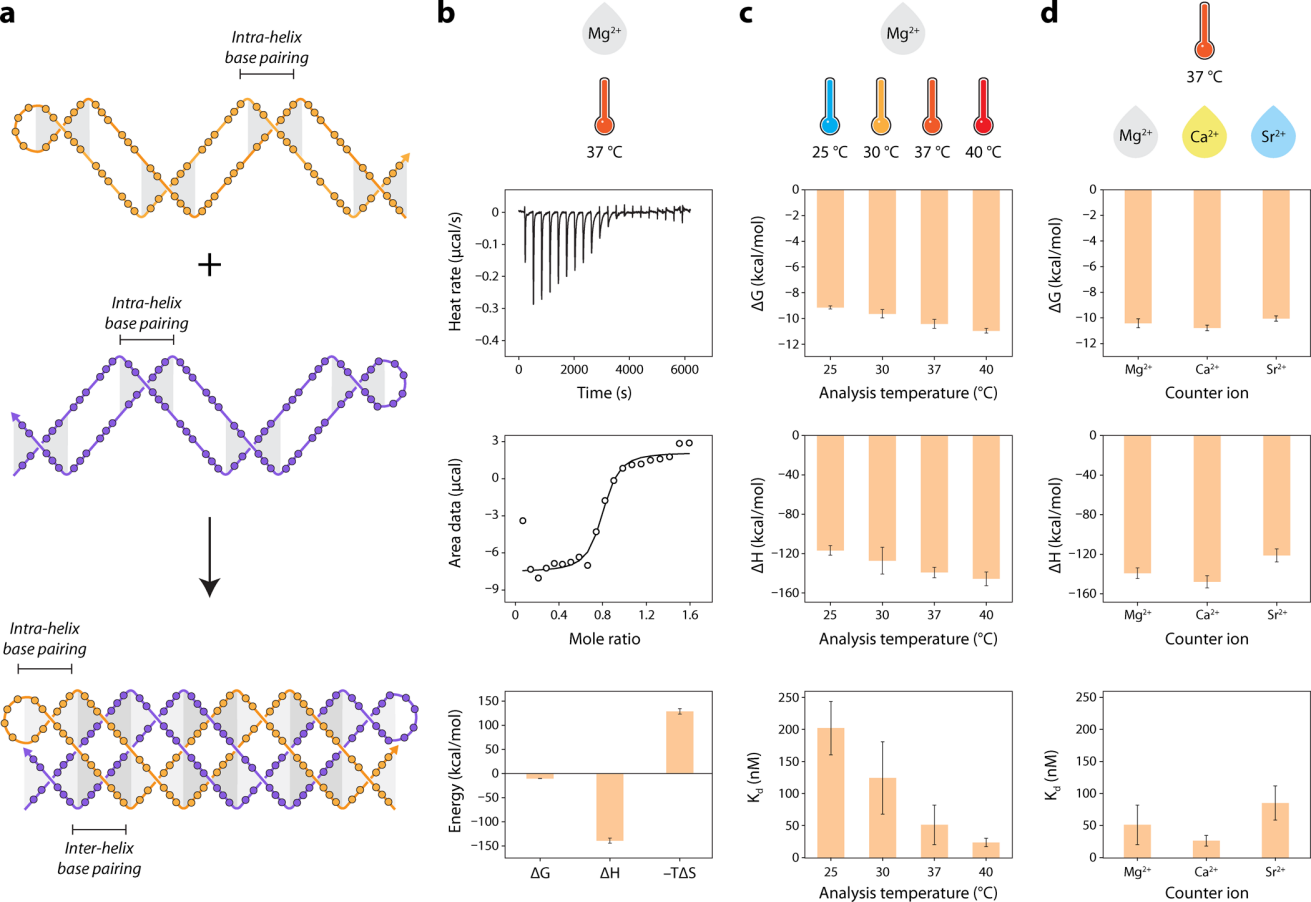
***Thermodynamics of the paranemic crossover (PX) assembly***. (a) Schematic showing the design of the PX assembly. (b) Isothermal titration calorimetry (ITC) thermograms and thermodynamic parameters of the PX assembly in 125 mM Mg^2+^ at 37 °C. (c) Comparison of thermodynamic parameters of PX assembly at different temperatures. (d) Comparison of thermodynamic parameters of PX assembly in different counterions at 125 mM ion concentration.

We chose 37 °C and buffer with 125 mM Mg^2+^ to derive the thermodynamic parameters for PX DNA. The PX assembly showed a ΔG of – 10.42 ± 0.35 kcal/mol with a ΔG per base pair of – 0.58 kcal/mol. Base-pairing between the half-PX molecules is enthalpically driven with a ΔH of – 139 ± 5 kcal/mol, which compensates for a substantial entropic penalty (ΔS = – 415 ± 18 cal/mol·K) (Figure 3b). Previous differential scanning calorimetry studies on unfolding of PX indicated ΔG at 37 °C to be ∼ 0.5 kcal/mol-bp for a PX DNA with 6:5 configuration.^32^

We then performed ITC at different temperatures to monitor the influence of the temperature on the thermodynamics of PX cohesion (Figure 3c and Figure S5–S6). The binding ratio of the molecules (n-value) increased from 0.70 ± 0.03 to 0.93 ± 0.07 as the temperature increased from 25 to 37 °C, which could be consistent with the interhelix hybridization of the half-PX molecules being favored at higher temperatures (Table S2). We observed a more favorable enthalpy when the temperature was increased from 25 to 40 °C (ΔΔH = ∼ 29 kcal/mol). This was accompanied by enthalpy–entropy compensation with a favorable change in ΔG from – 9.14 ± 0.12 to – 10.95 ± 0.18 kcal/mol suggesting that PX cohesion is favored by an increase in temperature. We determined the heat capacity (ΔC_p_) associated with PX cohesion to be – 1.9 kcal/mol·K from the temperature dependence of enthalpy (Figure S6). ΔC_p_ captures various aspects of nucleic acid folding, including conformational entropy, changes in solvent–solvent, and solvent–solute interactions.^33^ While it is difficult to completely unravel the molecular basis for heat capacity changes associated with PX formation, the observed ΔC_p_ of – 105 cal/mol·K per base pair could be attributed, at least in part, to changes in hydration during interhelix base-pairing of the PX molecules. Formation of PX may involve release of structured water and redistribution of counterions as the two partially folded duplexes wrap around each other and the unpaired surface-exposed bases in the half-PX molecules establish contiguous base stacks to form a rigid PX structure. Further, K_d_ decreased from 202 ± 42 nM at 25 °C to 23.5 ± 6.6 nM at 40 °C, indicating a higher affinity between the strands at higher temperatures. Following the ITC studies of the assemblies in buffer containing Mg^2+^, we performed the experiments in solutions containing Ca^2+^ and Sr^2+^ to study the impact of the counterions on the thermodynamics of the assembly (Figure 3d and Figure S7). The counterions had a relatively minor effect on thermodynamics, perhaps with the exception of Sr^2+^, which had less favorable binding both by ITC and by kinetics (Figure 2, Figure 3d, and Table S3).

A key advantage of the isothermal assembly of DNA nanostructures is the attachment of guest biomolecules at moderate temperatures. While several DNA nanostructures have been isothermally assembled and functionalized with model proteins, such as streptavidin, PX cohesion has not been employed to bridge two guest molecules. To demonstrate proof-of-concept for protein binding to isothermally assembled PX DNA, we incorporated a thrombin-binding aptamer sequence^34^ to the termini of each of the half-PX molecules (Figure 4a). In this design, the presence or absence of the aptamer sequence on each half of the PX DNA allows the controllable binding of one or two thrombin molecules on the structure at constant temperatures. Thrombin binding requires specific buffer conditions that include Na^+^ and K^+^ ions in addition to Mg^2+^. We first confirmed that the aptamer-modified PX molecules self-assembled isothermally with yields similar to those assembled by a thermal annealing protocol in this buffer (Figure S8). Nondenaturing gels showed retardation of the PX DNA band in the presence of thrombin, indicating successful binding of two thrombin molecules to the full PX structure, compared to only one thrombin bound to the PX structure containing one aptamer sequence (Figure 4b). In contrast, a PX structure lacking the aptamer sequence did not show any binding to thrombin (Figure S9).

**Figure 4:**
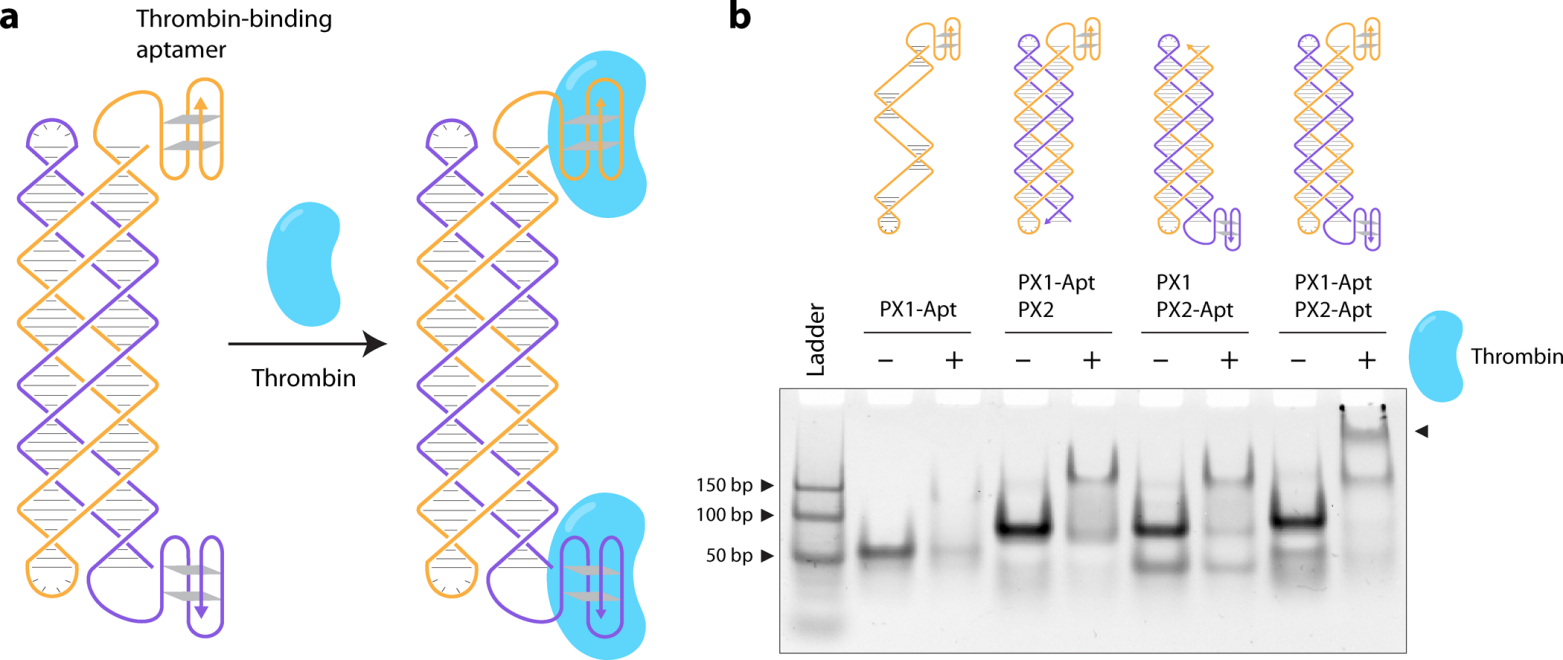
***Protein binding on isothermally assembled PX DNA***. (a) Schematic showing the design of PX containing a thrombin-binding aptamer. (b) Gel analysis showing the binding of thrombin to the isothermally assembled PX.

Overall, we show that isothermal paranemic cohesion of DNA molecules is possible in different counterions with a short incubation time (30–60 min) compared to the traditional 2-h or 2-day annealing protocols with minimal loss in assembly yields. Further, achieving this assembly at room temperature and physiological temperature makes this work adaptable to DNA nanotechnology applications in ambient environments. Our work also informs on the folding of long DNA segments involving multiple crossovers, with a crossover every half-turn in the case of PX DNA, as well as the effect of external factors such as the counterion concentration. Assembly under physiological conditions shown here, in combination with the exceptional biostability of PX molecules,^31,35^ could be useful in biological applications. A recent study showed isothermal assembly of DNA nanostructures in cell culture media and in the presence of living cells,^17^ indicating the potential of DNA nanostructure isothermal assembly in biological contexts. PX cohesion has been shown to be stronger than sticky-ended cohesion,^9^ and its efficiency at constant moderate temperatures can be used for assembly of topological constructs,^2,36^ 1D arrays, and 2D arrays^2,5^ and can be expanded to RNA-based construction.^37,38^ Our work extends this feature of PX cohesion to include attachment of protein guest molecules. Further, the reconfiguration of DNA nanostructures held by PX cohesion can be achieved at environmental temperatures using this strategy. Combined with prior work on strand displacement based PX devices^3,4^ and our earlier work on mismatch and enzyme based operation of PX devices,^39,40^ isothermal assembly allows both the construction and operation of such devices at constant temperatures.

## Supplementary Material



## Notes

Experimental procedures, additional results and DNA sequences used (PDF)

## References

[1] Shih W. M., Quispe J. D., Joyce G. F. (2004). A 1.7-Kilobase
Single-Stranded DNA
That Folds into a Nanoscale Octahedron. Nature.

[2] Ohayon Y. P., Sha R., Flint O., Chandrasekaran A. R., Abdallah H. O., Wang T., Wang X., Zhang X., Seeman N. C. (2015). Topological Linkage
of DNA Tiles Bonded by Paranemic Cohesion. ACS
Nano.

[3] Chakraborty B., Sha R., Seeman N. C. (2008). A DNA-Based Nanomechanical Device with Three Robust
States. Proc. Natl. Acad. Sci. U.S.A..

[4] Yan H., Zhang X., Shen Z., Seeman N. C. (2002). A Robust DNA Mechanical
Device Controlled by Hybridization Topology. Nature.

[5] Shen W., Liu Q., Ding B., Shen Z., Zhu C., Mao C. (2016). The Study
of the Paranemic Crossover (PX) Motif in the Context of Self-Assembly
of DNA 2D Crystals. Org. Biomol. Chem..

[6] Shen W., Liu Q., Ding B., Zhu C., Shen Z., Seeman N. C. (2017). Facilitation
of DNA Self-Assembly by Relieving the Torsional Strains between Building
Blocks. Org. Biomol. Chem..

[7] Han D., Qi X., Myhrvold C., Wang B., Dai M., Jiang S., Bates M., Liu Y., An B., Zhang F., Yan H., Yin P. (2017). Single-Stranded
DNA and RNA Origami. Science.

[8] Wang X., Chandrasekaran A. R., Shen Z., Ohayon Y. P., Wang T., Kizer M. E., Sha R., Mao C., Yan H., Zhang X., Liao S., Ding B., Chakraborty B., Jonoska N., Niu D., Gu H., Chao J., Gao X., Li Y., Ciengshin T., Seeman N. C. (2019). Paranemic Crossover
DNA: There and Back Again. Chem. Rev..

[9] Shen Z., Yan H., Wang T., Seeman N. C. (2004). Paranemic Crossover DNA: A Generalized
Holliday Structure with Applications in Nanotechnology. J. Am. Chem. Soc..

[10] Rodriguez A., Madhanagopal B. R., Sarkar K., Nowzari Z., Mathivanan J., Talbot H., Patel A., Morya V., Halvorsen K., Vangaveti S., Berglund J. A., Chandrasekaran A. R. (2025). Counterions
Influence the Isothermal Self-Assembly of DNA Nanostructures. Science Advances.

[11] Kopielski A., Schneider A., Csáki A., Fritzsche W. (2015). Isothermal
DNA Origami Folding: Avoiding Denaturing Conditions for One-Pot, Hybrid-Component
Annealing. Nanoscale.

[12] Jungmann R., Liedl T., Sobey T. L., Shih W., Simmel F. C. (2008). Isothermal
Assembly of DNA Origami Structures Using Denaturing Agents. J. Am. Chem. Soc..

[13] Zhang Z., Song J., Besenbacher F., Dong M., Gothelf K. V. (2013). Self-Assembly
of DNA Origami and Single-Stranded Tile Structures at Room Temperature. Angew. Chem., Int. Ed..

[14] Rossi-Gendron C., El Fakih F., Bourdon L., Nakazawa K., Finkel J., Triomphe N., Chocron L., Endo M., Sugiyama H., Bellot G., Morel M., Rudiuk S., Baigl D. (2023). Isothermal
Self-Assembly of Multicomponent and Evolutive DNA Nanostructures. Nat. Nanotechnol..

[15] Bourdon L., Afrose S. P., Agarwal S., Das D., Singh R., Di Cicco A., Lévy D., Yamada A., Baigl D., Franco E. (2025). Nanotubes Growth by
Self-Assembly of DNA Strands at
Room Temperature. ACS Nano.

[16] Chandrasekaran A. R. (2025). Isothermal
Assembly of DNA Nanostructures. Chem. Commun..

[17] Bourdon, L. ; Wilkens, G. D. ; Dehissi, S. ; Hotte, S. ; Rudiuk, S. ; Morel, M. ; Yamada, A. ; Bellot, G. ; Baigl, D. Ultra-Fast Isothermal Formation of DNA Nanostructures in Culture Media: Application to in Situ Assembly of DNA Origami with Living Cells. ChemRxiv,2025. 10.26434/chemrxiv-2025-mh3k7-v2. PMC12895221 41432148

[18] Madhanagopal B. R., Rodriguez A., Cordones M., Chandrasekaran A. R. (2024). Barium
Concentration-Dependent Anomalous Electrophoresis of Synthetic DNA
Motifs. ACS Appl. Bio Mater..

[19] Rodriguez A., Gandavadi D., Mathivanan J., Song T., Madhanagopal B. R., Talbot H., Sheng J., Wang X., Chandrasekaran A. R. (2023). Self-Assembly
of DNA Nanostructures in Different Cations. Small.

[20] Zhou K., Mei Z., Lei Y., Guan Z., Mao C., Li Y. (2022). Boosted Productivity
in Single-Tile-Based DNA Polyhedra Assembly by Simple Cation Replacement. ChemBioChem..

[21] Wang X., Zhang X., Mao C., Seeman N. C. (2010). Double-Stranded
DNA Homology Produces a Physical Signature. Proc. Natl. Acad. Sci. U.S.A..

[22] Gao X., Gethers M., Han S., Goddard W. A., Sha R., Cunningham R. P., Seeman N. C. (2019). The PX Motif of DNA Binds Specifically
to Escherichia Coli DNA Polymerase I. Biochemistry.

[23] Kizer M., Huntress I. D., Walcott B. D., Fraser K., Bystroff C., Wang X. (2019). Complex between a Multicrossover DNA Nanostructure, PX-DNA, and T7
Endonuclease I. Biochemistry.

[24] Lin C., Wang X., Liu Y., Seeman N. C., Yan H. (2007). Rolling Circle
Enzymatic Replication of a Complex Multi-Crossover DNA Nanostructure. J. Am. Chem. Soc..

[25] Lin C., Rinker S., Wang X., Liu Y., Seeman N. C., Yan H. (2008). In Vivo Cloning of Artificial DNA
Nanostructures. Proc. Natl. Acad. Sci. U.S.A..

[26] Maiti P. K., Pascal T. A., Vaidehi N., Goddard W. A. (2004). The Stability of
Seeman JX DNA Topoisomers of Paranemic Crossover (PX) Molecules as
a Function of Crossover Number. Nucleic Acids
Res..

[27] Maiti P. K., Pascal T. A., Vaidehi N., Heo J., Goddard W. A. (2006). Atomic-Level
Simulations of Seeman DNA Nanostructures: The Paranemic Crossover
in Salt Solution. Biophys. J..

[28] Naskar S., Maiti P. K. (2021). Mechanical Properties of DNA and DNA Nanostructures:
Comparison of Atomistic, Martini and oxDNA Models. J. Mater. Chem. B.

[29] Santosh M., Maiti P. K. (2011). Structural Rigidity
of Paranemic Crossover and Juxtapose
DNA Nanostructures. Biophys. J..

[30] Zhang X., Yan H., Shen Z., Seeman N. C. (2002). Paranemic Cohesion of Topologically-Closed
DNA Molecules. J. Am. Chem. Soc..

[31] Mandal, S. ; Chandrasekaran, A. R. ; Maiti, P. K. Mechanistic Insights into Crossover-Dependent Stability and Exceptional Resistance of PX/JX Nanostructures to DNase I Using Enhanced Sampling. bioRxiv, 2025. 10.1101/2025.04.17.649409.

[32] Spink C. H., Ding L., Yang Q., Sheardy R. D., Seeman N. C. (2009). Thermodynamics
of Forming a Parallel DNA Crossover. Biophys.
J..

[33] Mikulecky P. J., Feig A. L. (2006). Heat Capacity Changes Associated
with Nucleic Acid
Folding. Biopolymers.

[34] Dittmer W. U., Reuter A., Simmel F. C. (2004). A DNA-Based
Machine That Can Cyclically
Bind and Release Thrombin. Angew. Chem., Int.
Ed..

[35] Chandrasekaran A. R., Vilcapoma J., Dey P., Wong-Deyrup S. W., Dey B. K., Halvorsen K. (2020). Exceptional Nuclease Resistance of
Paranemic Crossover (PX) DNA and Crossover-Dependent Biostability
of DNA Motifs. J. Am. Chem. Soc..

[36] Topological bonding of DNA nanostructures - ProQuest. https://www.proquest.com/openview/ffc7c547d31feea4c86277a9d9b86fb3/1?pq-origsite=gscholar&cbl=18750 (accessed 2025-12-30).

[37] Afonin K.
A., Cieply D. J., Leontis N. B. (2008). Specific RNA Self-Assembly with Minimal
Paranemic Motifs. J. Am. Chem. Soc..

[38] Sampedro
Vallina N., McRae E. K. S., Geary C., Andersen E. S. (2023). An RNA
Paranemic Crossover Triangle as A 3D Module for Cotranscriptional
Nanoassembly. Small.

[39] Chandrasekaran A. R. (2024). A DNA Rotary
Nanodevice Operated by Enzyme-Initiated Strand Resetting. Chem. Commun..

[40] Talbot H., Chandrasekaran A. R. (2025). Mismatch-Induced
Toehold-Free Strand Displacement Used
to Control a DNA Nanodevice. ACS Synth. Biol..

