# Supplementary material for: Paranemic Cohesion of DNA under Isothermal Conditions
Source: JACS Au. 2026 Mar 6;6(4):2191–7. doi: 10.1021/jacsau.6c00130 (PMC13126193; doi:10.1021/jacsau.6c00130)
Supplement: Supplementary file 1 [file au6c00130_si_001.pdf]

Supporting information

Paranemic cohesion of DNA under isothermal conditions

Lauren A. Anderson,[1,#] Akul Patel,[1,#] Bharath Raj Madhanagopal[1,#] Hannah Talbot,[1,2] Nada Kabbara,[1] Ken Halvorsen,[3] and Arun Richard Chandrasekaran[1,3,*]

[1]Department of Nanoscale Science and Engineering, University at Albany, State University of New York, Albany, NY, USA.
[2]Department of Biological Sciences, University at Albany, State University of New York, Albany, NY, USA.
[3]The RNA Institute, University at Albany, State University of New York, Albany, NY, USA.

#Joint first authors
*Correspondence: arun@albany.edu

Methods

Preparation of DNA complexes.

DNA strands were purchased from Integrated DNA Technologies (IDT). For different metal ions, the chemicals used were magnesium acetate tetrahydrate, calcium chloride dihydrate, strontium chloride, lithium chloride, sodium chloride, potassium chloride and magnesium chloride (Sigma-Aldrich). Human α-thrombin was purchased from Haematologic Technologies Inc. For control DNA complexes annealed in Mg^{2+}, component DNA strands were mixed in TAE buffer containing 40 mM tris base (pH 8.0), 20 mM acetic acid, 2 mM EDTA, and 12.5 mM magnesium acetate (concentrations in 1× buffer). For samples annealed in other ions, component DNA strands were mixed in TAE buffer (1× final) containing the specified amounts of the corresponding metal salt. Control samples were annealed in a thermal cycler with the following steps: 90 °C for 3 minutes, 65 °C for 20 minutes, 45 °C for 20 minutes, 37 °C for 30 minutes, 20 °C for 30 minutes, and the solution was then cooled to 4 °C. For isothermal assembly, component DNA strands were mixed in 1× TAE containing the specified amounts of metal salt and incubated at 20 °C or 37 °C for 1 hour.

Non-denaturing polyacrylamide gel electrophoresis.

DNA samples were mixed with 10× loading dye containing bromophenol blue and glycerol and loaded in polyacrylamide gels (19:1 acrylamide solution, National Diagnostics) prepared in 1× TAE buffer containing 12.5 mM Mg^{2+}. Gels were typically run at 4 °C with 1× TAE-Mg^{2+} as the running buffer (except thrombin binding experiment, see later section) and stained with 0.5× GelRed (Biotium) in water for 20 min in dark and destained in water for 10 min. Gels were imaged on a Bio-Rad Gel Doc XR+ imager using the default settings for GelRed with UV illumination and analyzed using ImageLab software (Bio-Rad). Images were typically taken at multiple exposures to facilitate accurate quantification. For each gel, quantification was done using the highest-exposure image that did not contain saturated pixels in the bands. The assembly yield was quantified as the fraction of the intensity corresponding to the band of interest compared to the total intensity of all the bands in the lane. This yield was then normalized to the assembly yield of the structure prepared by annealing in 1× TAE buffer containing 12.5 mM Mg^{2+}.

Isothermal titration calorimetry.

For ITC experiments, strands PX1 and PX2 were prepared in 1x TA buffer containing 40 mM tris base (pH 8.0) and 20 mM acetic acid supplemented with 125 mM magnesium chloride, calcium chloride, or strontium chloride. The individual DNA strands were annealed by heating the solutions at 90 °C for 1 minute and cooling it to 20 °C over 35 minutes. All the solutions were degassed for 10 minutes prior to the titrations. The cell contained 5 μM of strand PX1, whereas the syringe contained 25 μM of strand PX2. All ITC experiments were conducted on an Affinity ITC instrument (TA instruments). In each titration, 2.5 μl of syringe solution was injected into the cell every 150 seconds with a total of 20 injections per titration. Titrations for PX strands were performed at 25 °C, 30 °C, 37 °C, and 40 °C. The heat of dilution was measured by titrating the respective syringe solution into cell containing only

the buffer and was subtracted from the sample data. The binding isotherm was fitted to the independent binding model, and the thermodynamic parameters were derived from the average of three titrations. Binding enthalpies (ΔH) were plotted as a function of temperature and fitted to a linear model to extract the slope, which yielded the change in heat capacity (ΔC_p).

Thrombin binding to PX with aptamer.

PX1-Apt and PX2-Apt strands containing thrombin binding aptamer sequence at the 3′ end of PX1 and PX2, respectively, were used to prepare PX-thrombin complexes. Stoichiometric amounts of the DNA strands were mixed in 1x TA buffer (pH 8.0) supplemented with 140 mM NaCl, 125 mM $MgCl_2$, and 10 mM KCl and incubated at 37 °C for 1 hour. Isothermally assembled PX with one or more thrombin binding aptamer domains were incubated with 2 molar equivalents of thrombin at 37 °C for 2 hours. Non-denaturing gels prepared and run using 0.5× tris-borate-EDTA buffer (pH 8.0) containing 12.5 mM $MgCl_2$ and 10 mM KCl were used to characterize the PX-thrombin complexes.

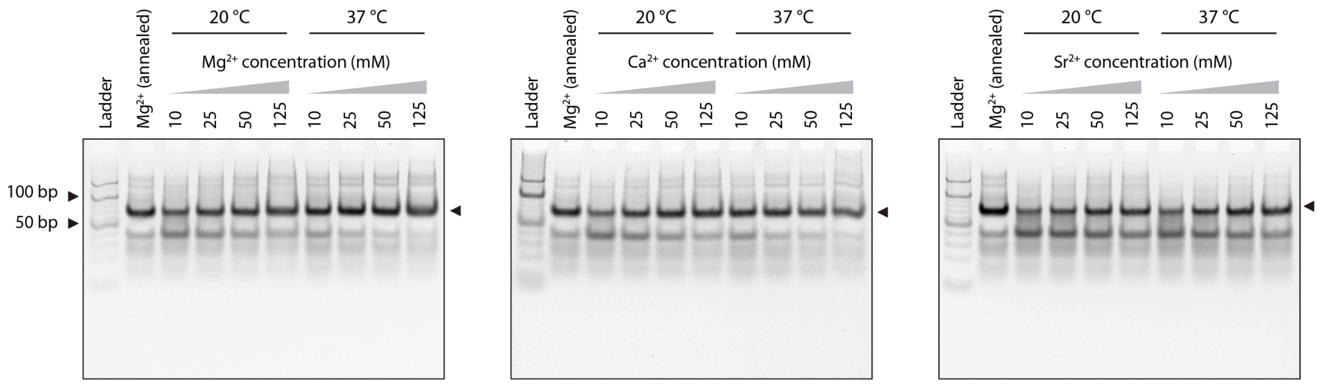

Figure S1. Isothermal assembly of PX DNA in TAE buffer containing different concentrations of Mg^{2+}, Ca^{2+} and Sr^{2+} at a constant temperature of 20 °C or 37 °C. Full images of gels shown in Figure 2b. Gels are representative images from experiments performed in triplicates.

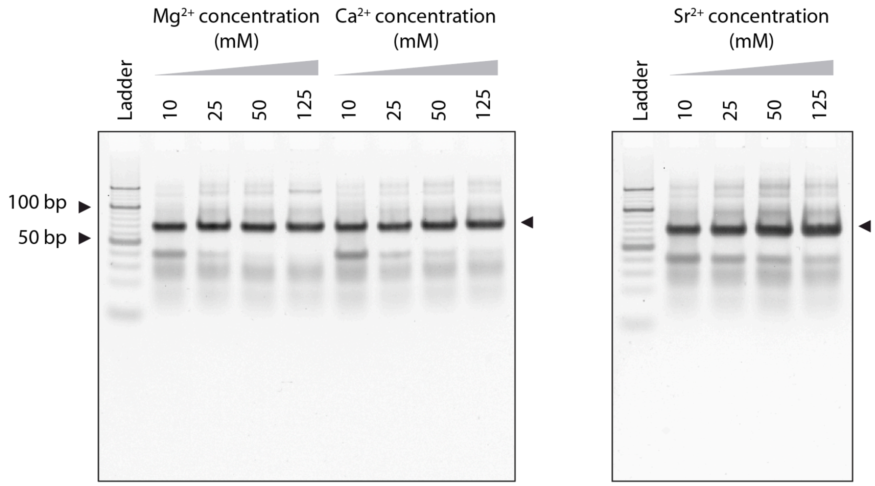

Figure S2. Assembly of PX DNA in TAE buffer containing different concentrations of Mg^{2+}, Ca^{2+} and Sr^{2+} using a thermal annealing protocol.

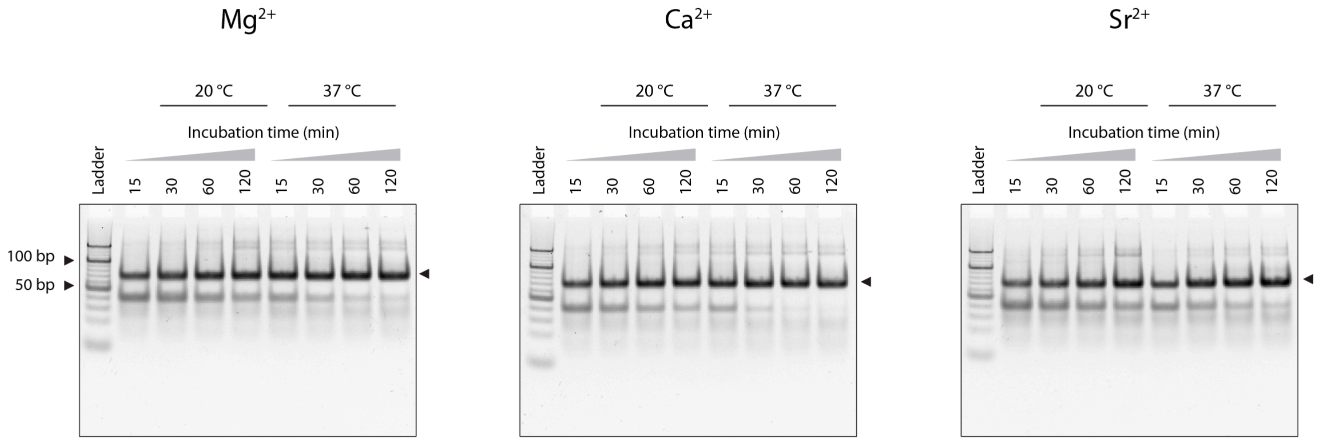

Figure S3. Time series for isothermal assembly of PX DNA in TAE buffer containing Mg^{2+}, Ca^{2+} or Sr^{2+} at a constant temperature of 20 °C or 37 °C. Full images of gels shown in Figure 2c. Gels are representative images from experiments performed in triplicates.

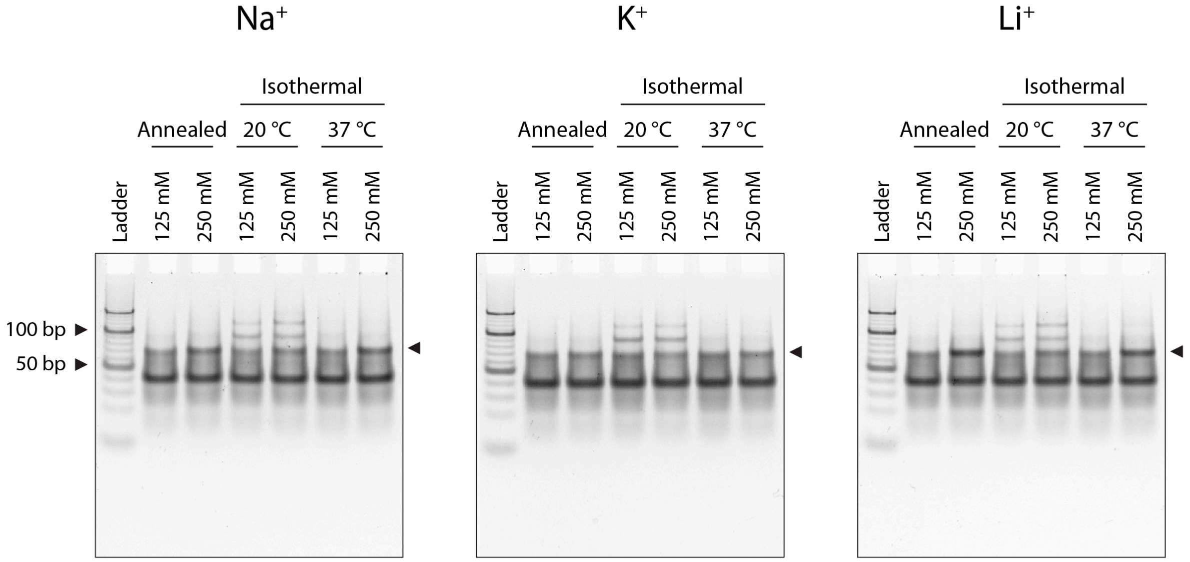

Figure S4. Isothermal assembly of PX DNA in TAE buffer containing different concentrations of Na^{+}, K^{+} or Li^{+} at a constant temperature of 20 °C or 37 °C.

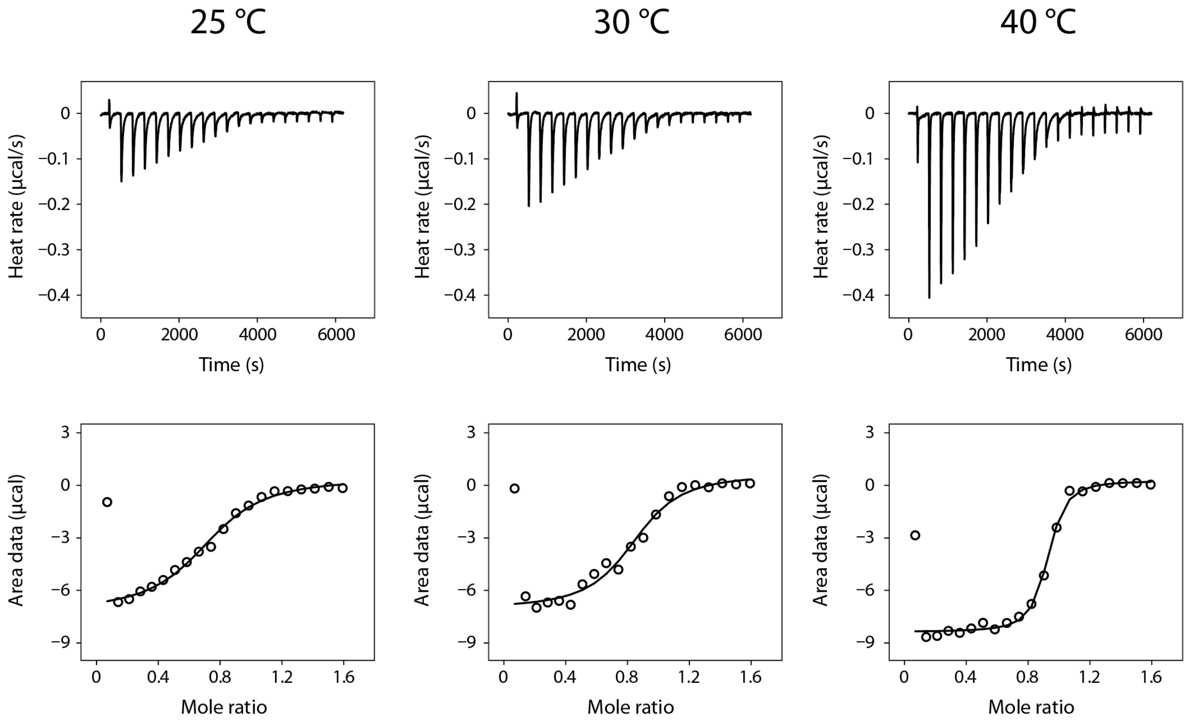

Figure S5. ITC thermograms of PX assembly in 1x TA containing 125 mM Mg^{2+} at different temperatures. Representative data from experiments performed in triplicates.

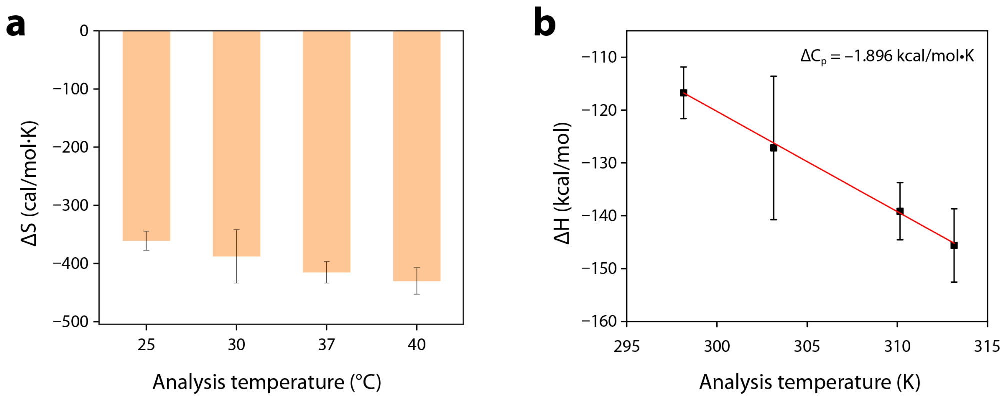

Figure S6. (a) ΔS of PX assembly at different temperatures and (b) heat capacity change (ΔC_p) of PX assembly.

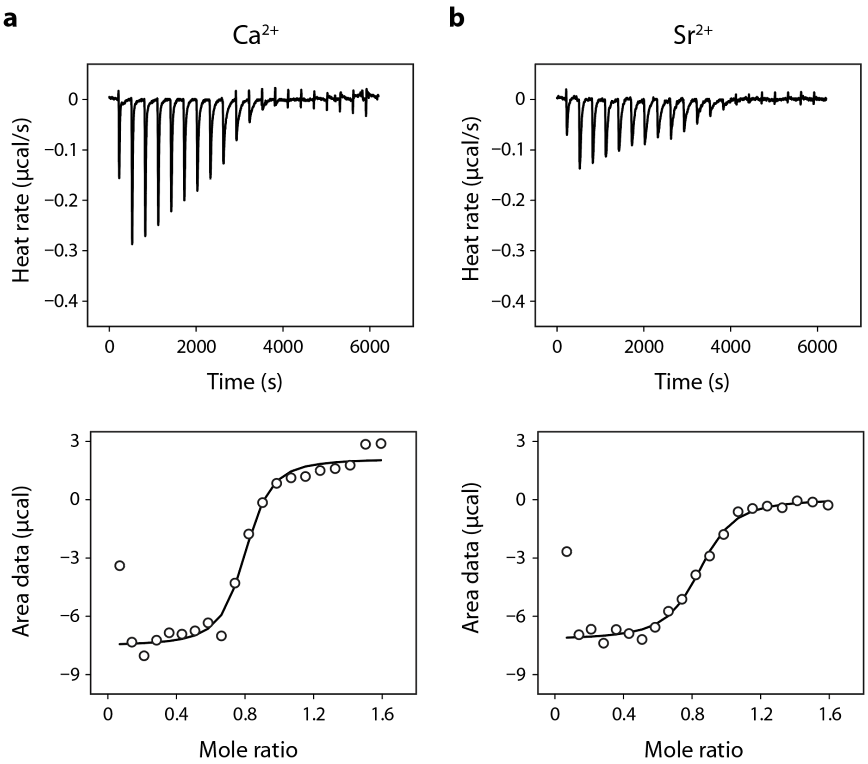

Figure S7. ITC thermogram and binding isotherm of paranemic cohesion in 1x TA containing 125 mM calcium chloride (a) and strontium chloride (b). Representative data from experiments performed in triplicates.

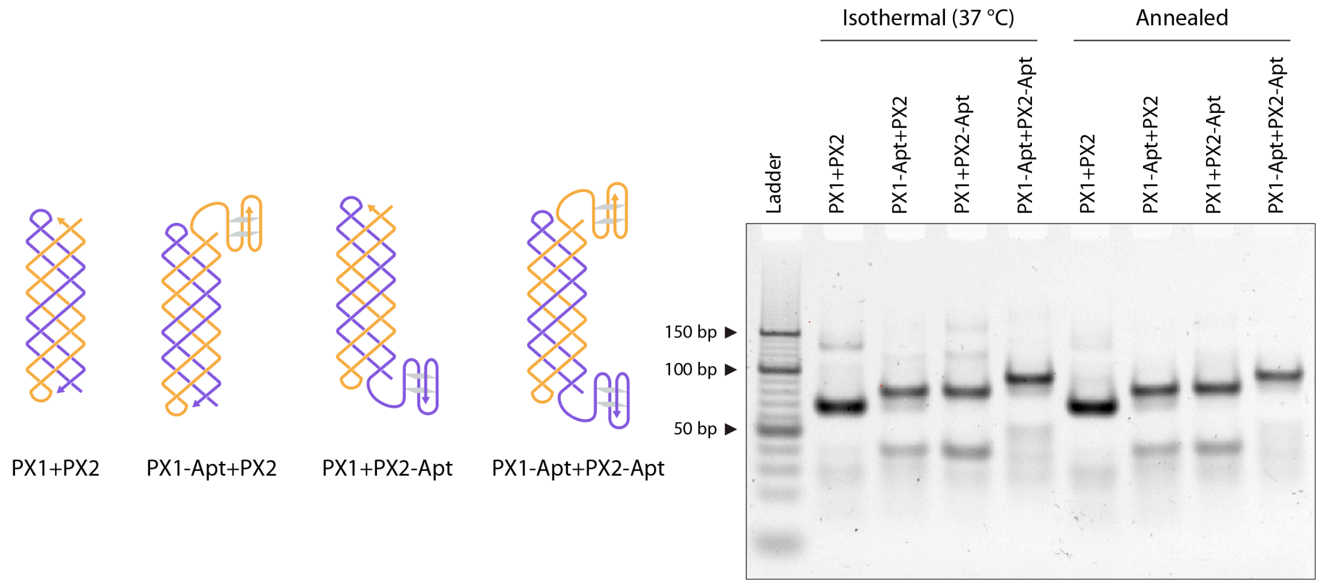

Figure S8. Isothermal assembly of PX-aptamer complexes compared to thermally annealed structures.

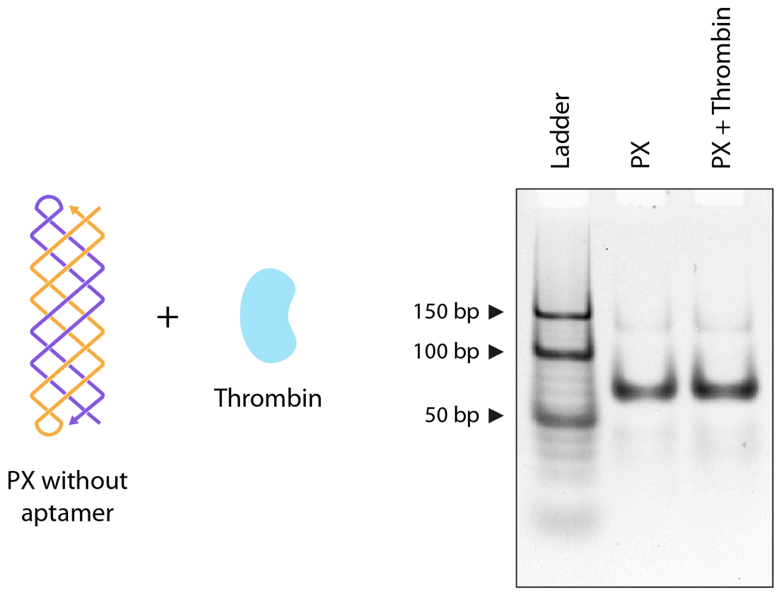

Figure S9. PX DNA without aptamer does not bind to thrombin.

Strand	Sequence (5′ to 3′)
PX1	CTCAGTTCGGTGCCTAATTGTGGCATTTGCGACACCACTTTTGTGGTATCATCAATGCTAT GTGTAGGCTTAGACCTGAG
PX2	ACTAGGTCGCAACAGACACAATACTTGACCGAATCACTTTTTAGTGAGTCTAACAAGTCA CATATCTGTGATGATCTAGT
PX1-Apt	CTCAGTTCGGTGCCTAATTGTGGCATTTGCGACACCACTTTTGTGGTATCATCAATGCTAT GTGTAGGCTTAGACCTGAG TTTT TGGTTGGTGTGGTTGGT
PX2-Apt	ACTAGGTCGCAACAGACACAATACTTGACCGAATCACTTTTTAGTGAGTCTAACAAGTCA CATATCTGTGATGATCTAGT TTTT TGGTTGGTGTGGTTGGT

Table S1. Sequences used in this study.

Temperature	n	K_D (nM)	ΔG (kcal/mol)	ΔH (kcal/mol)	ΔS (cal/mol.K)
25 °C	0.70 ± 0.03	202 ± 42	-9.14 ± 0.12	-117 ± 5	-361 ± 17
30 °C	0.82 ± 0.03	124 ± 57	-9.63 ± 0.32	-127 ± 14	-388 ± 46
37 °C	0.93 ± 0.07	51 ± 31	-10.42 ± 0.35	-139 ± 5	-415 ± 18
40 °C	0.88 ± 0.01	23.5 ± 6.6	-10.95 ± 0.18	-146 ± 7	-430 ± 23

Table S2. Thermodynamic parameters of PX DNA assembly in Mg^{2+}-containing buffer.

Counterion	n	K_D (nM)	ΔG (kcal/mol)	ΔH (kcal/mol)	ΔS (cal/mol.K)
Ca^{2+}	0.78 ± 0.01	26 ± 8	-10.78 ± 0.21	-148 ± 6	-442 ± 20
Sr^{2+}	0.83 ± 0.01	85 ± 27	-10.06 ± 0.21	-121 ± 7	-359 ± 22

Table S3. Thermodynamic parameters of PX assembly at 37 °C in buffer containing Ca^{2+} or Sr^{2+}.
